# Association of three single nucleotide polymorphisms in the LPIN1 gene with milk production traits in cows of the Yaroslavl breed

**DOI:** 10.18699/vjgb-24-14

**Published:** 2024-02

**Authors:** A.V. Igoshin, T.M. Mishakova, R.B. Aitnazarov, A.V. Ilina, D.M. Larkin, N.S. Yudin

**Affiliations:** Institute of Cytology and Genetics of the Siberian Branch of the Russian Academy of Sciences, Novosibirsk, Russia; Institute of Cytology and Genetics of the Siberian Branch of the Russian Academy of Sciences, Novosibirsk, Russia; Institute of Cytology and Genetics of the Siberian Branch of the Russian Academy of Sciences, Novosibirsk, Russia; Federal Williams Research Center for Forage Production and Agroecology, Scientific Research Institute of Livestock Breeding and Forage Production, Yaroslavl Region, Russia; Royal Veterinary College, University of London, London, United Kingdom; Institute of Cytology and Genetics of the Siberian Branch of the Russian Academy of Sciences, Novosibirsk, Russia

**Keywords:** cow, Yaroslavl breed, milk yield, fat percentage, protein percentage, fat yield, protein yield, LPIN1 gene, single nucleotide polymorphism, association, корова, ярославская порода, удой, процент жира, процент белка, выход жира, выход белка, ген LPIN1, однонуклеотидный полиморфизм, ассоциация

## Abstract

Lipin-1 is a member of the evolutionarily conserved family of proteins and is expressed predominantly in adipose tissue and skeletal muscle. On the one hand, lipin-1 is an enzyme that catalyzes the dephosphorylation of phosphatidic acid to diacylglycerol (DAG) and thus participates in the metabolic pathways of biosynthesis of storage lipids in the cell, membrane phospholipids, and intracellular signaling molecules. On the other hand, lipin-1 is able to be transported from the cytoplasm to the nucleus and is a coactivator of lipid metabolism gene transcription. It was shown, using the analysis of single nucleotide polymorphism (SNP) associations, that the lipin-1 coding gene (LPIN1) is a promising candidate gene for milk production traits in Holstein and Brown Swiss cows. However, it is unclear how much of its effect depends on the breed. The Yaroslavl dairy cattle breed was created in the 18–19 centuries in Russia by breeding northern Great Russian cattle, which were short and poor productive, but well adapted to local climatic conditions and bad food base. It was shown by whole genome genotyping and sequencing that the Yaroslavl breed has unique genetics compared to Russian and other cattle breeds. The aim of the study was to assess the frequency of alleles and genotypes of three SNPs in the LPIN1 gene and to study the association of these SNPs with milk production traits in Yaroslavl cows. Blood samples from 142 cows of the Yaroslavl breed were obtained from two farms in the Yaroslavl region. Genotyping of SNPs was carried out by polymerase chain reaction-restriction fragment length polymorphism method. Associations of SNPs with 305-day milk yield, fat yield, fat percentages, protein yield, and protein percentages were studied from the first to the fourth lactation. Statistical tests were carried out using a mixed linear model, taking into account the relationship between individuals. We identified three SNPs – rs110871255, rs207681322 and rs109039955 with a frequency of a rare allele of 0.042–0.261 in Yaroslavl cows. SNP rs110871255 was associated with fat yield during the third and fourth lactations. SNP rs207681322 was associated with milk yield for the second, third and fourth lactations, as well as protein yield for the third lactation. Thus, we identified significant associations of SNPs rs207681322 and rs110871255 in the LPIN1 gene with a number of milk production traits during several lactations in Yaroslavl cows.

## Abstract (ru)

Липин-1 является членом эволюционно-консервативного семейства белков и экспрессируется преимущественно в жировой ткани и скелетных мышцах. С одной стороны, липин-1 – это фермент, который дефосфорилирует фосфатидную кислоту до диацилглицерина и таким образом участвует в метаболических путях биосинтеза запасных липидов в клетке, фосфолипидов мембраны и внутриклеточных сигнальных молекул. С другой стороны, липин-1 способен транспортироваться из цитоплазмы в ядро и служит коактиватором транскрипции генов липидного метаболизма. С использованием анализа ассоциаций однонуклеотидных полиморфизмов (ОНП) было пока- зано, что ген липина-1 (LPIN1) является перспективным геном-кандидатом признаков молочной продуктивности у коров голштинской и бурой швицкой пород. Однако неясно, насколько его эффект зависит от породы. Ярославская молочная порода крупного рогатого скота была выведена в XVIII–XIX вв. в России путем разведения «в себе» северного великорусского скота, который был низкорослым и малопродуктивным, но хорошо адаптированным к местным климатическим условиям и скудной кормовой базе. С помощью полногеномного генотипирования и секвенирования было показано, что ярославская порода обладает уникальной генетикой по сравнению с российскими и зарубежными породами крупного рогатого скота. Целью работы была оценка частоты аллелей и генотипов трех ОНП в гене LPIN1 и исследование ассоциации этих ОНП с показателями молочной продуктивности у коров ярославской породы. Образцы крови от 142 коров ярославской породы были получены из двух хозяйств Ярославской области. Генотипирование ОНП выполняли методом анализа полиморфизма длин рестрикционных фрагментов после проведения полимеразной цепной реакции. Ассоциации ОНП с удоем, выходом молочного жира и белка, а также с процентным содержанием жира и белка в молоке за 305 дней лактации были исследованы с первой по четвертую лактацию. Для статистического анализа использовали смешанную линейную модель с учетом родства между индивидами. При исследовании коров ярославской породы выявили три ОНП: rs110871255, rs207681322 и rs109039955 с частотой редкого аллеля 0.042–0.261. ОНП rs110871255 был ассоциирован с выходом жира за третью и четвертую лактации, rs207681322 был ассоциирован с удоем за вторую, третью и четвертую лактации, а также с выходом белка за третью лактацию. Таким образом, мы выявили достоверные ассоциации ОНП rs207681322 и rs110871255 в гене LPIN1 с рядом показателей молочной продуктивности в ходе нескольких лактаций у коров ярославской породы.

## Introduction

The most important economic trait in dairy farming is cow milk productivity which includes milk, fat and protein yields, and fat and protein percentage (Gutierrez-Reinoso et al., 2021). All of these characteristics are complex quantitative traits controlled by a large number of genes having little impact on phenotype (Weller et al., 2017; Silpa et al., 2021; Bekele et al., 2023; Singh et al., 2023). Recently, such methods as quantitative trait loci (QTL) mapping, genome-wide association studies (GWAS) (Bekele et al., 2023; Chen S.Y. et al., 2023; Teng et al., 2023), targeted RNA sequencing (RNA-seq) (Fang et al., 2020; Ahmad et al., 2021) and detecting signatures of selection in genomes (Rajawat et al., 2022; Nayak et al., 2023; Persichilli et al., 2023) have been widely applied to identify the genes and mutations directly affecting milk yield and milk composition (Khatkar et al., 2004; Weller, Ron, 2011; Lopdell, 2023).

Previously, when analyzing the whole-genome genotyping (WGG) data, we identified selection signatures on chromosome 11 in a group of European dairy and dual-purpose (Bestuzhev, Holstein, Kholmogory, Black Pied, and Yaroslavl) cattle breeds, and the top SNP was localized in the lipin-1 (LPIN1) gene (Yurchenko et al., 2018a). Other authors also found selection signatures in this gene when analyzing the WGG data of the Yaroslavl and Holstein breeds (Zinovieva et al., 2020). However, our study did not detect any selection signatures in the LPIN1 gene region when analyzing the wholegenome sequencing data of the Yaroslavl breed (Ruvinskiy et al., 2022), which may be due to both a high significance threshold (q = 0.01) and a different set of breeds (Holstein, Kholmogory, Yakut) used for comparison.

Lipin-1 is a member of an evolutionarily conserved family that is represented by three proteins (lipins 1, 2, and 3) in most vertebrates (Csaki et al., 2013; Siniossoglou, 2013; Chen Y. et al., 2015; Saydakova et al., 2021). Lipin-1 is expressed predominantly in adipose tissues, skeletal muscles and, to a lesser extent, in the liver, brain and other tissues (Reue, Zhang, 2008). In one respect, it is a phosphohydrolase enzyme that dephosphorylates phosphatidic acid to diacylglycerol (DAG) and thus participates in the metabolic pathways for biosynthesis of cellular-storage lipids, membrane phospholipids, and intracellular signalling molecules. At the same time, lipin-1 is transported from the cytoplasm to the nucleus to coactivate lipid metabolism gene transcription. Although this protein lacks a DNA-binding domain, it has been shown to regulate transcription by interacting with other transcription factors (TF), e. g., it regulates adipocyte differentiation and functioning by interacting with the PPARgamma transcription factor (Kim et al., 2013), and fatty acid oxidation gene expression by interacting with the PPARalpha TF (Barroso et al., 2011). Also, lipin-1 binds to the mTORC1 protein complex and thus regulates the activity of the SREBP TF that, in turn, regulates multiple pathways for fatty acid, triglyceride and cholesterol biosynthesis (Peterson et al., 2011).

According to the NCBI Gene database, in cattle, the LPIN1 gene spans about 136 Kb on chromosome 11, consists of 25 exons, and encodes eight transcripts translated into proteins ranging in size from 895 to 1,010 amino acids (https://www. ncbi.nlm.nih.gov/gene/537224). LPIN1 mRNA expression in cow liver and mammary gland increases significantly at the peak of lactation compared to the beginning of lactation and dry periods (Bionaz, Loor, 2008; Li et al., 2020). Keeping lactating Holstein cows on a diet supplemented with fish and soybean oils to reduce milk fat yield caused an increase in LPIN1 mRNA expression in their subcutaneous adipose tissue (Thering et al., 2009). In vitro treatment of bovine mammary gland cells with rosiglitazone (BRL49653), a PPARgammaselective agonist, resulted in activation of LPIN1 mRNA expression (Kadegowda et al., 2009).

The rs137457402 and rs136905033 SNPs in this gene were associated with the content of five fatty acids in the milk of Brown Swiss cows (Pegolo et al., 2016). The rs137457402 SNP was also associated with the percentage of protein content in the milk of the same cows (Cecchinato et al., 2014). Han B. et al. (2019) showed the relationship of seven SNPs with milk yield, fat or protein percentage, and fat or protein yield in Chinese Holsteins, yet most of these associations were revealed only for the first or second lactations. Two nonsynonymous SNPs in the sixth exon of the LPIN1 gene were associated with fat and protein percentages in the milk of Holstein-Friesian×Jersey crossbred cows from New Zealand (Du et al., 2021). All of the above indicates that LPIN1 has been a promising candidate gene for milk productivity traits, but it is unclear to what extent its effect is breed-specific.

The Yaroslavl dairy breed was created in the 18–19th centuries in Russia in Yaroslavl Province (Dmitriev, 1978; Dmitriev, Ernst, 1989; Dunin, Dankvert, 2013; Stolpovsky et al., 2022). The animals are mostly black, with the head, belly, lower limbs and tip of the tail being white. They have characteristic black glasses-like markings around the eyes (see the Figure). The breed was created based on inter-se mating of Northern Great Russian cattle which was stunted and unproductive but well adapted to local climatic conditions and poor forage. The initial selection was based on the exterior and then on milk yield and fat percentage. In the 19–20th centuries, the Yaroslavl breed was crossbred with the Tyrolean, Angeln, Simmental, Algauz, Jersey, Dutch and Kholmogory cattle. In the USSR, crossbreeding with the Ostfriesian and Holstein bulls was also carried out. However, these crosses are believed to have had a small impact, as the Yaroslavl cattle have retained their specific exterior (Dmitriev, 1978; Dmitriev, Ernst, 1989; Stolpovsky et al., 2022).

**Fig. 1.**
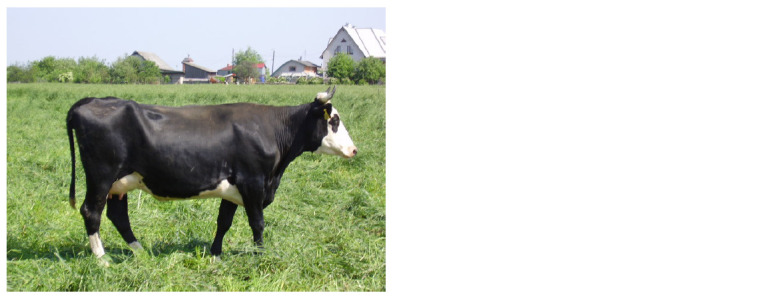
Yaroslavl cattle.

In 2022, the total breed population was about 30,000 animals that are characterized by high milk yield (6,590 kg for 305 lactation days) and fat percentage (4.13 %) (Shichkin et al., 2023). WGG (Iso-Touru et al., 2016) and microsatellite analysis (Abdelmanova et al., 2020) have demonstrated that the Yaroslavl breed has unique genetic parameters compared to Russian and foreign livestock, and foreign breeds have had a minor impact on the Yaroslavl cattle’s gene pool (Sermyagin et al., 2018; Yurchenko et al., 2018b; Zinovieva et al., 2020).

The purpose of the present study was to estimate the allele and genotype frequencies of three SNPs in the LPIN1 gene and to study the association of these SNPs with milk productivity traits in Yaroslavl cows.

## Materials and methods

As a material for the study, blood samples from 142 Yaroslavl cows from two farms of the Yaroslavl Region were used. The phenotypic data extracted from breeding record cards were provided by the Selex Information and Analytical System.

DNA extraction was performed using the standard phenolchloroform extraction method with preliminary proteolytic treatment (Sambrook, Russell, 2006). Genotyping of the rs110871255, rs109039955, and rs207681322 SNPs in the LPIN1 gene was carried out by restriction fragment length polymorphism (RFLP) analysis after polymerase chain reaction (PCR). Primers were designed using the Vector NTI software package (Lu, Moriyama, 2004). The specificity of each primer pair was evaluated in silico using the primer-BLAST algorithm (Ye et al., 2012). The primers, PCR reaction conditions and restriction enzymes are given in Supplementary Material 11. The test for deviation from Hardy–Weinberg equilibrium and linkage disequilibrium between the studied SNPs (LD) were calculated in PLINK v1.9 (--ld option) (Purcell et al., 2007). For this purpose, the genotypic data were converted to the PED format recognized by the program


Supplementary Materials are available in the online version of the paper: https://vavilov.elpub.ru/jour/manager/files/Suppl_Igoshin_Engl_28_1.pdf


The studied associations included those related to such traits as milk, milk fat and protein yields as well as milk fat and protein percentages for 305 lactation days. Data from four lactations were included in the analysis. If a cow’s lactation period was less than 305 days, its milk, fat and protein yields for this period were standardized to 305 days by the formula (Wiggans, Van Vleck, 1979):

**Formula. 1.**
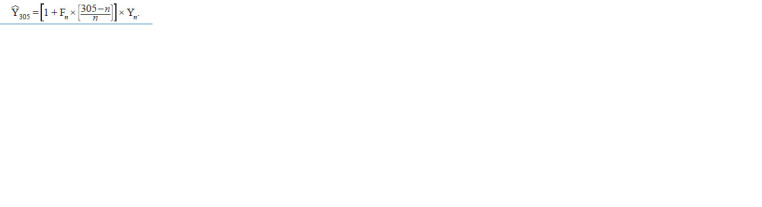
Formula. 1

Here, Y305 is the expected productivity for 305 days; n is the actual lactation duration in days; Yn is the productivity for the actual lactation period; Fn is Shook’s factor for the n-th day to account for the decrease in productivity during lactation (Hillers, Williams, 1981). This factor is calculated based on the productivity data for the animals with lactation duration ≥ 305 days as

**Formula. 2.**
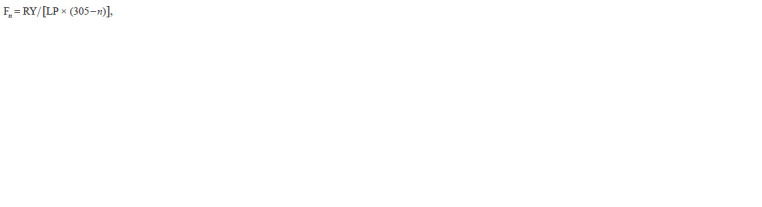
Formula. 2

where RY is the difference between the 305-day productivity and that for n days; and LP is the productivity at the n-th day. The daily data required to calculate the corresponding values were derived from cumulative productivity curves (Supplementary Material 2). The protein and fat percentages for incomplete lactations were calculated from the data standardized to 305 days.

Statistical analysis was performed using the mixed linear model implemented in the “lme4qtl” R package (“relmatLmer” function) (Ziyatdinov et al., 2018). This model allows one to account for genetic relationships between individuals by modelling polygenic effects based on a kinship matrix. The matrix was calculated using the “kinship2” R package (“kinship” function) (Sinnwell et al., 2014), based on the animal pedigree data from their breeding record cards. Genotypes were coded as 0, 1, and 2 implying the additive contribution of SNP alleles to a trait. The cows’ birth and calving (one preceding the lactation period) seasons were used as additional predictors. Since the error in calculating the expected milk yield for 305 days was probably higher for shorter lactations, the analysis was performed using the “weights” option of the “relmatLmer” function. For lactation periods of 305 days or more, a weight of 305 was taken, and for shorter durations, one equal to the actual number of lactation days. The Benjamini–Hochberg method (Benjamini, Hochberg, 1995) implemented in the “qvalue” R package (the “qvalue” function with “lambda=0” parameter) was applied to correct for multiple comparisons (Storey et al., 2023).

## Results

Target fragments were successfully amplified for 136 (rs109039955), 130 (rs207681322) and 109 (rs110871255) animals. All three studied SNPs were found to be polymorphic in the studied sample (Table 1). The genotype distributions of rs207681322 and rs110871255 deviated significantly from Hardy–Weinberg equilibrium ( p = 0.0141 and p = 0.0039, respectively), and that of rs109039955 did not. The LD between the studied SNPs was r2 = 0.098, D′ = 0.853 for rs109039955 and rs207681322; r2 = 0.003, D′ = 0.061 for rs109039955 and rs110871255; r2 = 0.001, D′ = 0.088 for rs207681322 and rs110871255.

**Table 1.**
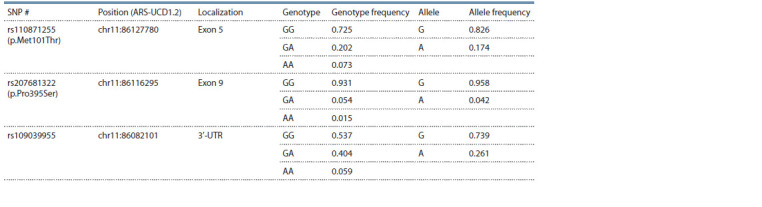
Characteristics of the genotyped SNPs

Statistical tests revealed a total of six associations of two SNPs with three milk production traits from the 2nd to 4th lactations (Table 2). No associations of all three SNPs were detected for the first lactation. For the second lactation, one SNP (rs207681322) was associated with milk yield (q = 0.043). For the third lactation period, one SNP (rs110871255) was associated with fat yield (q = 0.0135) and another (rs207681322) with milk (q = 2.54E–04) and protein (q = 0.021) yields. For the fourth period, one SNP (rs110871255) was associated with fat yield (q = 0.0348) and another (rs207681322) with milk yield (q = 0.021).

**Table 2.**
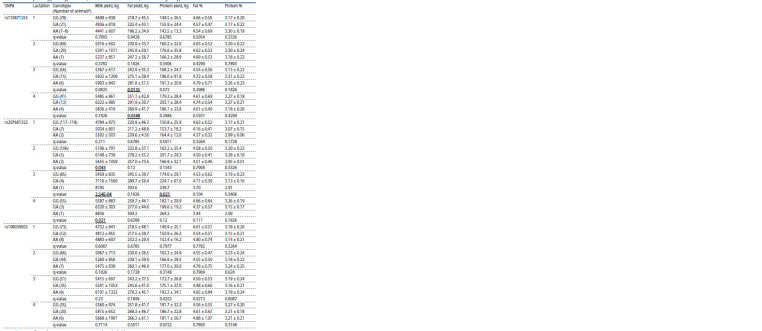
Associations of the studied SNPs with milk productivity traits from 1st to 4th lactations and their corresponding phenotypic values (mean ± standard deviation) for different genotypes The statistically significant associations (q <0.05) are indicated in bold. * Only animals having respective phenotypic data were accounted for. The markedly lower number of animals genotyped for rs110871255 was due to the limited amount of DNA available for analysis.

Therefore, rs110871255 was associated with fat yield during the third and fourth lactations, and rs207681322 with milk yield in the second, third and fourth lactations as well as with protein yield in the third lactation.

## Discussion

In the studied Yaroslavl cows, three SNPs (rs110871255, rs207681322 and rs10903995) with a rare allele frequency of 0.042–0.261 were identified. For the rs207681322 and rs110871255 loci, genotype distributions differed significantly from those expected by Hardy–Weinberg equilibrium due to an excess of rare homozygotes, which may be due to inbreeding, gene drift, or selection in farm animal populations (Hedrick, 2005). Such deviations are often observed in studies of microsatellite DNA markers or SNPs in different cattle breeds (Melka, Schenkel, 2012; Madilindi et al., 2020; Ocampo et al., 2021). It is noteworthy that tests for Hardy–Weinberg equilibrium deviation are used to verify random mating in populations, while tests for deviations from expected homozygote frequency are performed to estimate inbreeding coefficients (Haldane, 1984; Robertson, Hill, 1984). The LD between the studied SNPs in Yaroslavl cows differs significantly from that described previously for the same SNPs in Chinese Holstein cows (Han B. et al., 2019), but these patterns are known to vary significantly between cattle breeds (Porto-Neto et al., 2014).

For two SNPs, we found significant associations with such traits as milk, fat and protein yields across multiple lactations, e. g., allele A of rs207681322 had a positive effect on the cows’ milk and protein yields. Previously, these associations were found in the first or second lactation in Chinese Holsteins (Han B. et al., 2019). In our study, the А allele of rs110871255 positively affected fat yield. However, in Holstein cows, the same allele was associated with increased fat percentage during the second lactation period (Han B. et al., 2019).

Unlike Han B. et al. (2019), the associations we found were in subsequent lactations rather than in the first one, which may be due to both genetic (different breeds) and environmental (different breeding conditions) factors. It should be emphasized that there is still no consensus on the effect hereditary factors have on milk productivity during lactation. While some authors believe that the heritability of these traits in the Holstein breed for the first lactation is higher than for subsequent ones (Dimov et al., 1995; Yamazaki et al., 2016; Lee et al., 2020), the latest large-scale study of nearly 3.5 million records from over 1 million Holstein cows found increased heritability of milk, fat and protein yields specifically in lactations 3 and 4 compared to lactations 1 and 2 (Williams et al., 2022), which agrees well with our results. Milk yield heritability for individual test days has been significantly higher during the third lactation compared to the first lactation in holsteinized native cattle from Thailand (Buaban et al., 2020) and Sahiwal cows from Kenya (Ilatsia et al., 2007).

The associations we found correspond well with the data of other authors on the effect LPIN1 has on lactation, lipid biosynthesis and general metabolic levels. The gene’s mRNA expression has significantly increased during lactation in the mammary gland of humans (Mohammad, Haymond, 2013), pigs (Lv et al., 2015) and mice (Han L.Q. et al., 2010). SNP in the LPIN1 gene is associated with the percentage of visceral and intramuscular fat in pigs (He et al., 2009). The gene’s mutation leading to the expression of a truncated protein causes lipodystrophy in rats (Mul et al., 2011). The FLD mice with mutated LPIN1 have a phenotype similar to that of hereditary lipodystrophy in humans characterized by subcutaneous fat loss, hepatic steatosis, insulin resistance, etc. (Péterfy et al., 2001). Conversely, LPIN1 overexpression in adipose tissues or skeletal muscles causes obesity in transgenic mice (Phan, Reue, 2005). Moreover, while LPIN1 expression in adipose tissue stimulates adipocytes to accumulate fat, in muscle tissue its expression affects the whole body’s energy expenditure (temperature; food and oxygen consumption). Suppression of LPIN1 expression in muscle cells in vitro has led to insulin resistance (Huang et al., 2017). At the same time, a negative correlation was observed between LPIN1 mRNA levels in adipose tissue and blood glucose/insulin concentrations in humans and mice (Suviolahti et al., 2006). The same authors found the SNPs in the LPIN1 gene are associated with blood insulin levels in dyslipidemia families (Suviolahti et al., 2006). In their opinion, LPIN1 plays an essential role in glucose homeostasis and its genetic variants affect metabolism traits. Indeed, genetic variations of the gene are associated with the development of some metabolic syndromes in humans (Brahe et al., 2013; Zhang et al., 2013).

## Conclusion

Our study has found the rs207681322 and rs110871255 SNPs in the LPIN1 gene have statistically significant associations with some milk productivity traits during several lactations in the Yaroslavl cows to be previously found by other authors in the Holstein breed. The results obtained can be used in markerassisted and genomic selection for dairy cattle breeding.

## Conflict of interest

The authors declare no conflict of interest.
